# Quantifying and correcting slide-to-slide variation in multiplexed immunofluorescence images

**DOI:** 10.1093/bioinformatics/btab877

**Published:** 2022-01-04

**Authors:** Coleman R Harris, Eliot T McKinley, Joseph T Roland, Qi Liu, Martha J Shrubsole, Ken S Lau, Robert J Coffey, Julia Wrobel, Simon N Vandekar

**Affiliations:** Department of Biostatistics, Vanderbilt University Medical Center, Nashville, TN 37203, USA; Epithelial Biology Center, Vanderbilt University Medical Center, Nashville, TN 37232, USA; Department of Cell and Developmental Biology, Vanderbilt University School of Medicine, Nashville, TN 37232, USA; Epithelial Biology Center, Vanderbilt University Medical Center, Nashville, TN 37232, USA; Department of Surgery, Vanderbilt University School of Medicine, Nashville, TN 37232, USA; Department of Biostatistics, Vanderbilt University Medical Center, Nashville, TN 37203, USA; Center for Quantitative Sciences, Vanderbilt University Medical Center, Nashville, TN 37232, USA; Division of Epidemiology, Vanderbilt Ingram Cancer Center, Vanderbilt University Medical Center, Nashville, TN 37232, USA; Epithelial Biology Center, Vanderbilt University Medical Center, Nashville, TN 37232, USA; Department of Cell and Developmental Biology, Vanderbilt University School of Medicine, Nashville, TN 37232, USA; Epithelial Biology Center, Vanderbilt University Medical Center, Nashville, TN 37232, USA; Division of Gastroenterology, Hepatology, and Nutrition, Department of Medicine, Vanderbilt University Medical Center, Nashville, TN 37232, USA; Department of Biostatistics & Informatics, Colorado School of Public Health, Aurora, CO 80045, USA; Department of Biostatistics, Vanderbilt University Medical Center, Nashville, TN 37203, USA

## Abstract

**Motivation:**

Multiplexed imaging is a nascent single-cell assay with a complex data structure susceptible to technical variability that disrupts inference. These *in situ* methods are valuable in understanding cell–cell interactions, but few standardized processing steps or normalization techniques of multiplexed imaging data are available.

**Results:**

We implement and compare data transformations and normalization algorithms in multiplexed imaging data. Our methods adapt the ComBat and functional data registration methods to remove slide effects in this domain, and we present an evaluation framework to compare the proposed approaches. We present clear slide-to-slide variation in the raw, unadjusted data and show that many of the proposed normalization methods reduce this variation while preserving and improving the biological signal. Furthermore, we find that dividing multiplexed imaging data by its slide mean, and the functional data registration methods, perform the best under our proposed evaluation framework. In summary, this approach provides a foundation for better data quality and evaluation criteria in multiplexed imaging.

**Availability and implementation:**

Source code is provided at: https://github.com/statimagcoll/MultiplexedNormalization and an R package to implement these methods is available here: https://github.com/ColemanRHarris/mxnorm.

**Supplementary information:**

Supplementary data are available at *Bioinformatics* online.

## 1 Introduction

Single-cell assays are increasingly valued for their ability to provide information about the cell micro-environment and cell population interactions in healthy and cancerous tissues (Islam *et al.*, 2020; McKinley *et al.*, 2019; Shrubsole *et al.*, 2008). Multiplexed imaging methods such as multiplexed immunofluorescence (MxIF) (Gerdes *et al.*, 2013), multiplexed immunohistochemistry (IHC) (Tsujikawa *et al.*, 2017) and CODEX (Goltsev *et al.*, 2018) are *in situ* analyses of multiple marker channels over a large number of cells within a given tissue sample. These methods build upon dissociative single-cell analysis methods like flow cytometry (Bradford *et al.*, 2004) and single-cell RNA sequencing (Chen *et al.*, 2019) to allow scientists to better understand spatial cell–cell interactions in biological samples. 

One significant issue in multiplexed imaging data is the presence of systematic noise at a variety of levels, related to batch and slide effects, imaging variables and optical effects (Berry *et al.*, 2021; Chang *et al.*, 2020). A single experiment may contain hundreds of slides and terabytes of data across which a researcher seeks to make inference (Maric *et al.*, 2021). However, this data complexity and the within-slide dependencies induce complex effects that can disrupt inference. This technical variability can be compounded through the complex image pre-processing pipeline and may contribute to biases that increase type 1 or type 2 error. Furthermore, it is difficult to develop a standardized pre-processing pipeline because of substantial variability in the markers used across different studies, as target proteins differ across organs and cancer types (Schapiro *et al.*, 2021; Yapp *et al.*, 2021).

Image normalization is a technique used to adjust the input pixel- or image-level values of an image to remove noise and improve image quality. Due to the nascent development of multiplexed imaging, there are few established statistical tools that address challenges related to technical variation in this dataset (Chang *et al.*, 2020). Normalization methods may improve similarity across images by removing the unknown effect of technical variability. Moreover, statistical methods for batch correction and image normalization can be modified to fit this complex data structure to ultimately reduce systematic noise and improve statistical inference.

Extensive work has been done in other fields to adjust for batch effects and systematic noise, particularly with regards to neuroimaging and genetic sequencing data. One primary method employed in both of these fields is the ComBat method, introduced for genetic micro-array data (Johnson *et al.*, 2007) and then adapted to neuroimaging in the analysis of magnetic resonance imaging (MRI) data (Fortin *et al.*, 2017; Yu *et al.*, 2018). The ComBat method is a location-scale model that implements an empirical Bayes algorithm to adjust for batch effects and is robust to outliers in small sample sizes. Curve registration, a non-parametric tool from functional data analysis (FDA), has been used in recent work to adjust for systematic variability in accelerometry and MRI data (Marron *et al.*, 2015; Wrobel *et al.*, 2020, 2019). In the neuroimaging context, curve registration is used to normalize the imaging data by non-linearly transform the image intensity domain so that it is similar across images from different subjects, potentially collected on different scanners. Multiplexed imaging data are further complicated because it is non-negative, which other groups have remarked upon in similar imaging applications like spatial transcriptomics (Elosua-Bayes *et al.*, 2021)—this requires unique derivations and/or applications of normalization methods to ensure no contradictions arise from negative marker intensities.

While adaptable, existing methods for normalizing data from other domains cannot be directly applied within multiplexed imaging due to the unusual format of the data (cell populations can differ substantially across samples), and the heavy skewness of the image histogram. The few algorithms adapted specifically for normalizing multiplex imaging data still could benefit from upstream normalization using algorithms adapted from other domains (Chang *et al.*, 2020; Raza *et al.*, 2016). For example, the RESTORE algorithm is a method developed for multiplexed imaging that uses negative control cells to remove unwanted variation across slides (Chang *et al.*, 2020). However, this method relies on clustering mutually exclusive marker pairs using cell-level labels that are defined using unnormalized marker intensities and thus embed biases as detailed in this article. Raza *et al.* (2016) also introduced normalization methods in the multiplexed imaging that implement a procedure of image filters and transformations. These methods show improvements at the pixel and image level, but do not correct for slide or batch effects that are prevalent as detailed in this work. Hence, the normalization methods proposed here can be applied early in the image processing pipeline to reduce bias in subsequent steps like phenotyping and spatial correlation analyses.

In this article, we introduce and compare normalization and data transformation methods for multiplexed imaging data. These techniques combine transformations of the scale of the data from its raw form with algorithms (namely, ComBat and functional data registration) adapted to remove slide effects from the data. We further develop multiple novel metrics to quantify and measure the removal of technical variation in these data, where cell populations can differ across slides. We use data from the Human Tumor Atlas Network to evaluate the methods we compare here (Rozenblatt-Rosen *et al.*, 2020). While we apply the methods here to segmented and quantified single-cell data from multiplexed imaging, they can also be applied at the pixel level.

## 2 Materials and methods

### 2.1 Implementation

We compare three data transformations: $\;{\text{log}}_{10}$, mean division (division by the slide-level mean) and mean division with $\;{\text{log}}_{10}$, and three normalization procedures: no normalization, ComBat and functional data registration, for a total of nine potential multiplex image normalization algorithms (Table 1).

**Table 1.** Summary of normalization procedures implemented

	None	ComBat	Registration (fda)
$\;{\text{log}}_{10}$	$\;{\text{log}}_{10}(y+1)$	$\;\text{ComBat}\left(\;{\text{log}}_{10}(y+1)\right)$	$\;\text{fda}\left(\;{\text{log}}_{10}(y+1)\right)$
Mean division	$\frac{y}{\mu_{ic}}$	$\;\text{ComBat}\left(\frac{y}{\mu_{ic}}\right)$	$\;\text{fda}\left(\frac{y}{\mu_{ic}}\right)$
$\text{Mean division}\;\;{\text{log}}_{10}$	$\;{\text{log}}_{10}(\frac{y}{\mu_{ic}}+\frac{1}{2})$	$\;\text{ComBat}\left(\;{\text{log}}_{10}(\frac{y}{\mu_{ic}}+\frac{1}{2})\right)$	$\;\text{fda}\left(\;{\text{log}}_{10}(\frac{y}{\mu_{ic}}+\frac{1}{2})\right)$

*Note*: Transformations (rows) and normalization (columns) performed on the data. Here, *y* is the median cell intensity values for an arbitrary marker channel *c*, and *μ_ic_* is the slide mean for slide *i* of the median cell intensity values for marker channel *c*.

#### 2.1.1 Transformations

Let $Y_{ic}(u)$ denote the raw intensity of unit *u* on slide *i* for marker channel *c* (here *u* corresponds to segmented cell intensities). We consider the following transformations: the $\;{\text{log}}_{10}$ transformation, $\;{\text{log}}_{10}(Y_{ic}(u)+1)$, where the addition of 1 follows since $Y_{ic}(u)$ is integer-valued; the mean division transformation: $\frac{Y_{ic}(u)}{\mu_{ic}}$, where *μ_ic_* is the mean intensity value on slide *i* for channel *c*; and the mean division $\;{\text{log}}_{10}$ transformation, $\;{\text{log}}_{10}\left(\frac{Y_{ic}(u)}{\mu_{ic}}+\frac{1}{2}\right)$, where again *μ_ic_* is the mean intensity value on slide *i* for channel *c*. Here, the data are no longer integer-valued, and the addition of $\frac{1}{2}$ ensures values greater than $\frac{1}{2}$ are positive and less than $\frac{1}{2}$ are negative to properly adjust this scale of data. Other transformations, including a Z-score transformation, can be found in the Supplementary Material.

#### 2.1.2 Combat normalization

We adapted the empirical Bayes framework of the ComBat algorithm (Fortin *et al.*, 2017; Johnson *et al.*, 2007) for multiplexed imaging data. We parameterize mean and variance of the slide-level batch effects, with the location-scale model

$$Y_{ic}(u)=\alpha_{c}+\gamma_{ic}+\delta_{ic}\varepsilon_{ic}(u),$$

where we define $Y_{ic}(u)$ as the intensity of unit *u* on slide *i* for marker channel *c* and *α_c_* as the grand mean of $Y_{ic}(u)$ for channel *c*. Though in principle, units can be at the pixel or cell level, in our application, $Y_{ic}(u)$ is the median cell intensity (or its transformed counterpart) of a selected marker for a given segmented cell on a specific slide in the dataset. Here *γ_ic_* is the mean batch effect of slide *i* for channel *c* and we assume $\gamma_{ic}∼N(\gamma_{c},\tau_{c}^{2}),\;\delta_{ic}^{2}$ is the variance batch effect of slide *i* for channel *c* and we assume $\delta_{ic}^{2}∼IG(\omega_{c},\beta_{c})$, and we assume the random errors $\varepsilon_{ic}(u)∼N(0,1)$. We use the data to estimate ${\hat{\alpha }}_{c}$ and then estimate ${\hat{\gamma }}_{ic}=\frac{1}{U_{ic}}\sum_{u}Y_{ic}(u)$, or the sample mean intensity on slide *i* for channel *c*. We further define ${\hat{\sigma }}_{c}=\frac{1}{N}\sum_{ic}(Y_{ic}(u)-{\hat{\alpha }}_{c}-{\hat{\gamma }}_{ic}{)}^{2}$ and let:

$$Z_{ic}(u)=\frac{Y_{ic}(u)-{\hat{\alpha }}_{c}}{{\hat{\sigma }}_{c}^{2}},$$

where we assume $Z_{ic}(u)∼N(\gamma_{ic},\delta_{ic}^{2})$. Based on the posterior conditional means, we find the following empirical Bayes estimators of the two batch effect parameters (a detailed derivation of these estimators can be found in the Supplementary Material):

$$\delta_{ic}^{2*}=\frac{{\bar{\beta }}_{c}+\frac{1}{2}\sum_{u}(Z_{ic}(u)-\gamma_{ic}^{*}{)}^{2}}{\frac{U_{ic}}{2}+{\bar{\omega }}_{c}-1},\gamma_{ic}^{*}=\frac{U_{ic}\cdot {\bar{\tau }}_{c}^{2}\cdot {\hat{\gamma }}_{ic}+\delta_{ic}^{2*}\cdot {\bar{\gamma }}_{c}}{U_{ic}\cdot {\bar{\tau }}_{c}^{2}+\delta_{ic}^{2*}}$$

Where we define *U_ic_* as the number of quantified cells present on a particular slide *i* for a given channel *c*. We calculate the hyper-parameter estimates of ${\bar{\beta }}_{c},{\bar{\omega }}_{c},{\bar{\tau }}_{c}^{2},{\bar{\gamma }}_{c}$ using the method of moments and iterate between estimating the hyper-parameters and batch effect parameters until convergence (Dempster *et al.*, 1977; Johnson *et al.*, 2007). Upon convergence, we use these batch effects to adjust the data,

$$Y_{ic}^{*}(u)=\frac{{\hat{\sigma }}_{c}^{2}}{{\hat{\delta }}_{ic}^{*}}(Z_{ic}(u)-{\hat{\gamma }}_{ic}^{*})+{\hat{\alpha }}_{c}.$$

This model adjusts the Z-normalized intensity data, $Z_{ic}(u)$, by the mean and variance batch effects, and re-scales back to the initial scale of the data with the mean and variance of the raw marker intensity values. Note that zeroes were left in the data prior to the ComBat normalization, since for each scale transformation we perform on the data the zeroes are meaningful rather than an absence of signal.

#### 2.1.3 Functional data registration

For the second normalization algorithm, we implemented functional data registration using the fda R package (Ramsay *et al.*, 2020; Ramsay and Silverman, 2005). This approach uses FDA methods to approximate the histograms for each slide and channel as smooth densities, and uses functional registration to align the densities to their average at the slide-level. Functional registration is performed by estimating a monotonic warping function for each density that stretches and compresses the intensities such that densities are aligned. These warping functions are then used to transform the marker intensity values in the images so that non-biological variability is reduced across slides.

Here, let our observed cell intensity values $Y_{ic}(u)$ have density $Y_{ic}(u)∼f(y|i,c)$. Our goal is to remove technical variation related to the slide by estimating a warping function, $\phi_{ic}(y)$, which is a monotonic transformation of the intensities. We first use a 21 degree of freedom cubic B-spline basis to approximate the densities of the median cell intensities for each slide and marker, $f(y|i,c)\approx \beta^{T}g(y)$ where $\beta \in {\mathbb{R}}^{21}$ is an unknown coefficient vector and *g*(*y*) is a vector of known basis functions. We then register the approximated histograms to the average, restricting the warping function to be a 2 degree of freedom linear B-spline basis for some functions $h_{1}(y)$ and $h_{2}(y)$ and for constants *C*_0_ and *C*_1_ to be estimated from the data,

$$\phi_{ic}(x)=C_{0}+C_{1}\int_{0}^{x}\;\text{exp}\;\left\{\beta_{1ic}h_{1}(y)+\beta_{2ic}h_{2}(y)\right\}dy,$$

such that the transformation is monotonic (Ramsay and Silverman, 2005). Unknown parameters $\beta_{1ic}$ and $\beta_{2ic}$ are estimated to minimize,

$$\int_{y}\|f_{ic}(\phi_{ic}(y))-f(y){\|}^{2}dy$$

Where *f*(*y*) is the average density across slides. We then use $\phi_{ic}(y)$ to calculate the normalized intensity values, $Y_{ic}^{*}(u)$:

$$Y_{ic}^{*}(u)=\phi_{ic}(Y_{ic}(u))$$

Note that the warping function $\phi_{ic}(y)$ is a map that takes in the raw median cell intensity value and outputs a new, normalized intensity value. Images are then normalized by taking the original intensity values in the image, and transforming them using the map defined by the warping function. This combined process can be summarized as first taking the raw data, smoothing the histogram of these data using a B-spline basis expansion, and then calculating a warping function to transform the smoothed data so that densities across slides within marker channel *c* are approximately aligned.

### 2.2 Evaluation framework

There is no accepted gold standard for evaluating normalization methods in multiplexed imaging because the same tissue sample cannot be imaged twice and there is substantial heterogeneity across samples (Nadarajan *et al.*, 2019; Rozenblatt-Rosen *et al.*, 2020). Here, our evaluation framework relies on the two following conditions to be deemed successful: (i) reduction in slide-to-slide variance in the cell intensity data and (ii) preservation (and potential improvement) of existing biological signal in the data.

#### 2.2.1 Alignment of marker densities

To determine if between-slide noise is visible when comparing densities, we visually inspect the changes in density curves for each transformation method. *A priori*, we expect that a successful transformation method will align the density curves across slides, and subsequently we inspect the placement of slide-level Otsu thresholds, a commonly used thresholding algorithm used in imaging analysis (Otsu, 1979), to confirm a reduction in variability between slides. To quantitatively measure the alignment of marker densities, we implement the *k*-sample Anderson–Darling statistic to quantify the likelihood that each slide is drawn from the same population (Scholz and Stephens, 1987). A higher value of this test statistic indicates greater evidence that the *k*-samples are drawn from different distributions.

#### 2.2.2 Threshold discordance and accuracy

Otsu thresholding is a commonly used thresholding algorithm that defines an optimal threshold in gray-scale images and histograms, maximizing the between-class variance of pixel values to separate the data into two classes (Otsu, 1979). In this use case, we define Otsu thresholds at the slide-level for each of the markers in the study, where a cell with intensity value greater than the Otsu threshold is deemed marker positive. We then compare this to a global Otsu threshold, combining all slides, for each marker to calculate a mean discordance score across all slides for a given marker. For some marker channel *c*, slide *i*, and set of marker intensity values $Y_{ic}(u)$, define the indicator function for a given Otsu threshold *o* as $O_{ic}(u,o)=I(Y_{ic}(u)>o)$. Here, $O_{ic}(u,o)$ indicates which cells are in the expressed category using threshold *o*. The discordance metric is then defined as:

$$\frac{1}{N}\sum_{i}^{N}\left(\frac{\sum_{y}|O_{ic}(u,o_{ic})-O_{c}(u,o_{c})|}{U_{ic}}\right)$$

Where *U_ic_* is the number of quantified cells present on a particular slide *i* for a given channel *c*, *o_ic_* is the slide and channel-specific Otsu threshold, and *o_c_* is the threshold estimated across all slides for a given channel. Here we calculate a slide-level discordance score, e.g. the proportion of cells misclassified on each slide, and take an average of the score across slides for each marker channel. This measures the slide-to-slide discordance across all markers and transformation methods, to determine how similar Otsu thresholds are across slides following transformation. In this framework, a lower value of the threshold discordance score indicates better agreement across slides in the data.

We further implemented Otsu thresholding across slides to compare definitions of a marker positive cell with the manual labels of CD3 and CD8 as marker positive cells (see Section 2.3). This metric quantifies the accuracy of the Otsu thresholding method in recapitulating the bronze standard labels for each transformation method.

#### 2.2.3 Proportions of variance

To further assess the removal of slide-related variance following each transformation of the data, we fit a random effects model using the lme4 R package (Bates *et al.*, 2015) with a random intercept for slide to assess what proportion of variance is present at the slide-level for each marker. A successful normalization algorithm will reduce the slide-level variance, ultimately removing technical variability to improve the quality of the data.

#### 2.2.4 UMAP embedding

The Uniform Manifold Approximation and Projection (UMAP) is a technique for dimension reduction (McInnes *et al.*, 2018) commonly used in the biological sciences to distinguish differences in cell populations between single-cell data (Becht *et al.*, 2019). Here we reduce the data into two UMAP embeddings for each of the transformation methods using only four markers in the dataset: vimentin, collagen, pan-cytokeratin and ${\text{Na}}^{+}$/$K^{+}$-ATPase. These markers were chosen for their ability to easily distinguish epithelial and stromal cells. We expect the UMAP embeddings to yield clear separation of the data when using the epithelium label in our dataset (see Section 2.3). To quantify this separation of groups, we implement a k-means clustering model on the UMAP embeddings to predict the class label, and use the adjusted Rand index to measure the similarity with the true labels (Hartigan and Wong, 1979; Hubert and Arabie, 1985). Larger values of this index indicate better agreement between two sets of labels, adjusted for the chance grouping of elements. Note that across each slide in the dataset, approximately 10% of the data was used to derive the UMAP embeddings to reduce computational and visualization time.

### 2.3 Dataset

The data were collected from human colorectal cancer tissue samples from the Human Tumor Atlas Network (Rozenblatt-Rosen *et al.*, 2020). The final dataset comprises over 2.2 million cells in the MxIF modality across over 2400 images on 43 different slides, with single-cell segmentation performed using an algorithm developed in-house (McKinley *et al.*, 2019). Cell intensities for each marker were quantified as the median pixel value within the segmented cell, with tissue samples stained for 33 different marker channels. For the purpose of evaluating the algorithms compared in the article, we restricted our attention to the following markers: beta catenin (BCATENIN), CD3D (CD3), CD8 (CD8), collagen (COLLAGEN), ${\text{Na}}^{+}$/$K^{+}$-ATPase (NAKATPASE), olfactomedin 4 (OLFM4), pan-cytokeratin (PANCK), SRY-Box 9 (SOX9) and vimentin (VIMENTIN). These markers were chosen because of their ability to distinguish between epithelial and stromal cells, PANCK, COLLAGEN, NAKATPASE and VIMENTIN (Blom *et al.*, 2017; Ijsselsteijn *et al.*, 2019); as immune markers, CD3, CD8 (Galon *et al.*, 2006); as stem cell markers, OLFM4, SOX9 (Scott *et al.*, 2010; Van der Flier *et al.*, 2009); and as implicated in colon cancer, BCATENIN (Shang *et al.*, 2017).

We used epithelial and stromal cell labels and manually labeled marker positive cells as biological variables in order to quantify loss or improvement of biological signal due to each normalization method. The epithelial labels were created for each slide at the image level using a random forest trained on all of the markers included in the dataset (for a complete list, see the Supplementary Material). A cell was labeled as being in a particular cell class if that was the most likely class probability within the segmented cell area. We defined marker positive cells by first manually thresholding the immune marker images to create marker positive image masks. Then, for each segmented cell, the cell was defined as marker positive if more than 30% of its area contained marker-positive pixels. We refer to these as manual labels for CD3 and CD8. We also used a tumor image mask to denote whether a cell is in a tumor-containing region.

## 3 Results

### 3.1 Removal of slide-to-slide variation

#### 3.1.1 Alignment of marker densities

**Fig. 1.**
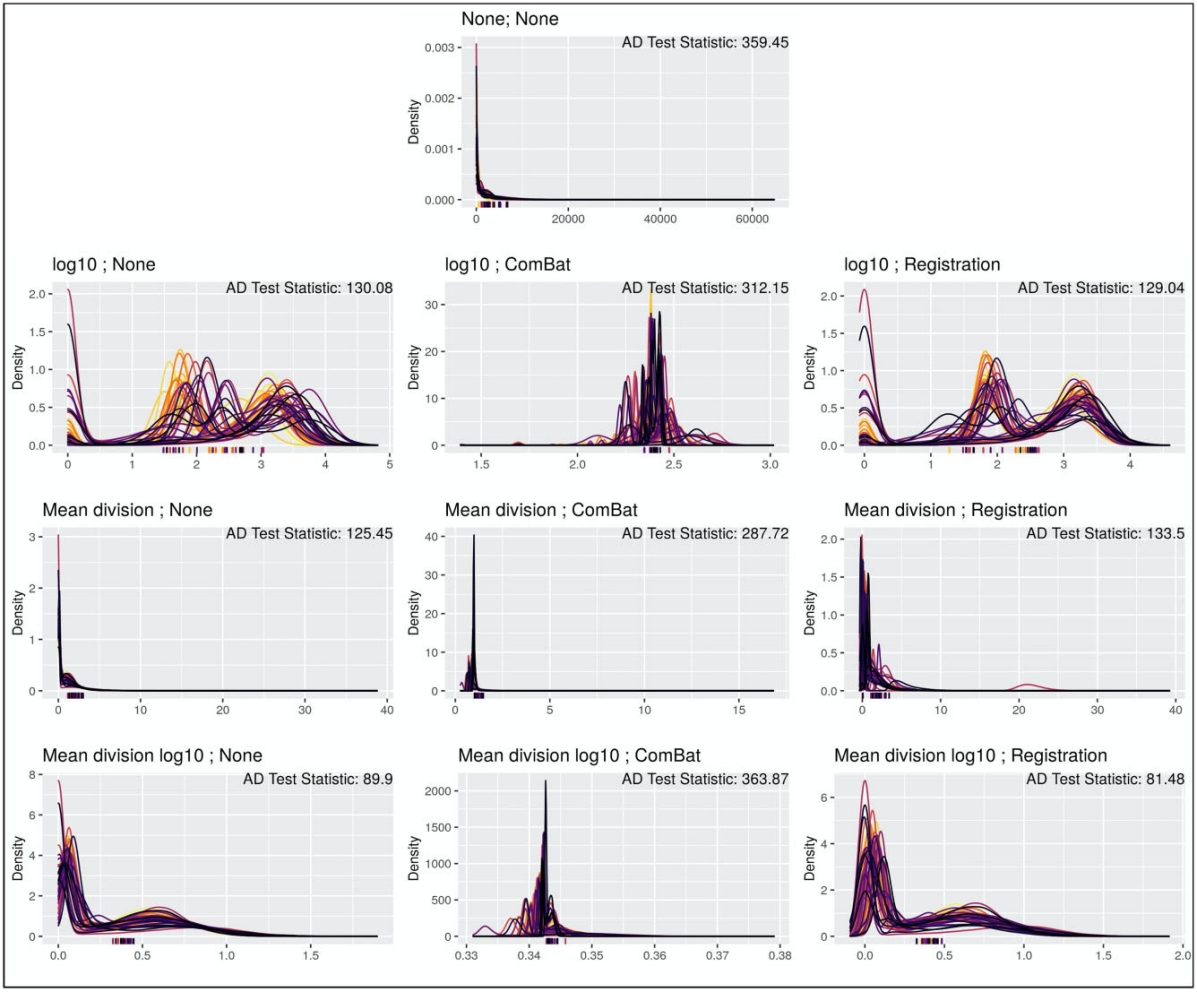
Visual comparison of vimentin marker densities for each transformation method. Density plots for the median cell intensity of the marker vimentin, where each color represents a different slide in the dataset. Each row is aligned with the scale transformations present in Table 1, where each column also matches with the normalization algorithms in Table 1. The ticks on the *x*-axis represent the Otsu thresholds for each slide for that transformed data, where the color again corresponds to the slide (such that the colors are one-to-one between threshold and density plot). Anderson–Darling test statistics for the marker vimentin are presented for each method in the top right corner

Density curves of the marker vimentin for each transformation algorithm and corresponding slide-level Otsu thresholds, along with test statistics from the *k*-sample Anderson–Darling test were compared to determine alignment of curves across slides after transformation (Fig. 1, Table 2). Beginning with the unnormalized transformed values, the $\;{\text{log}}_{10}$ transformation produces density curves that are somewhat well-aligned (AD Test: 130.08), while the mean division and mean division $\;{\text{log}}_{10}$ methods both compress the scale of the data and align well across slides (AD Test: 125.45, 89.90). Furthermore, each ComBat method performs poorly at aligning and reducing noise in the data, yielding the largest statistics from the Anderson–Darling test and visually noisy density curves. This is likely due to the Gaussian assumptions of the ComBat model that are not met in either the bi-modal ($\;{\text{log}}_{10}$, mean division $\;{\text{log}}_{10}$) or right-skewed (mean division) methods. The functional data registration aligns the $\;{\text{log}}_{10}$ and mean division $\;{\text{log}}_{10}$ well, and the algorithm yields marginal improvements for some of these transformations.

**Table 2.** Quantitative metrics comparing normalization methods

Method	Mean AD test statistic	Mean Otsu discordance score	Adj. Rand index (slide ID)	Mean variance proportion (slide ID)
None; None	275.019	0.085	0.033	0.138
$\;{\text{log}}_{10}$ ; None	225.413	0.134	0.083	0.301
$\;{\text{log}}_{10}$ ; ComBat	291.900	0.138	0.089	0.000
$\;{\text{log}}_{10}$ ; Registration	217.649	0.110	0.037	0.232
Mean division; None	138.774	0.041	0.007	0.000
Mean division; ComBat	247.612	0.109	0.064	0.000
Mean division; Registration	174.933	0.164	0.120	0.333
Mean division $\;{\text{log}}_{10}$; None	114.653	0.055	0.010	0.046
Mean division $\;{\text{log}}_{10}$; ComBat	321.810	0.132	0.071	0.000
Mean division $\;{\text{log}}_{10}$; Registration	104.330	0.049	0.018	0.081

*Note*: Results from the *k*-samples Anderson–Darling test statistic, the threshold discordance score, and the variance proportion at the slide level from the random effects modeling, all averaged across marker channels, as well as the adjusted Rand index for the slide identifiers comparing the raw data to the normalized data. For each of these metrics, small values indicate better performance for a given method.

The best performing methods for this metric are the mean division, mean division $\;{\text{log}}_{10}$, and mean division $\;{\text{log}}_{10}$ combined with the functional data registration algorithm: the data is well-aligned across slides and when averaging Anderson–Darling statistics across all marker channels (Table 2), we see these methods yield the lowest values presenting stronger evidence these values are derived from the same parent distribution. We also compared density curves of the markers CD3 and CD8 for each transformation algorithm, which largely present the same results (Supplementary Figs S1 and S2).

#### 3.1.2 Threshold discordance score

**Fig. 2.**
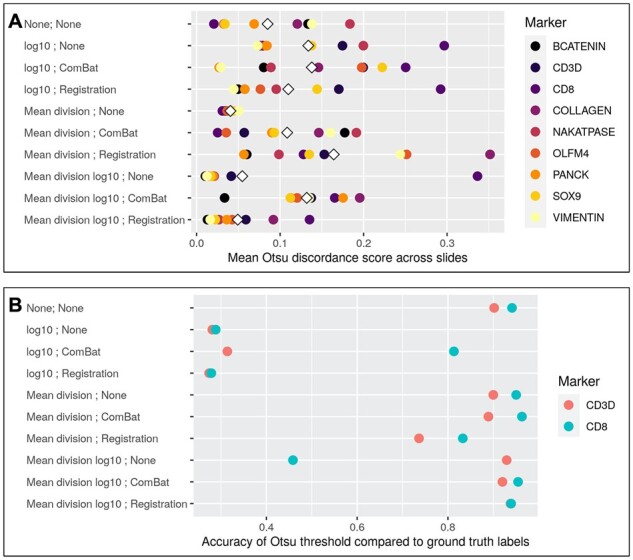
Threshold discordance and accuracy. (**A**) Otsu thresholds were calculated at the slide-level for each marker and compared to a global Otsu threshold for each marker to calculate a discordance score to compare transformation methods. The mean difference of the slide-level Otsu thresholds and the global Otsu threshold is then calculated for each marker, and presented as a point for each of the nine markers, with the white diamond representing the mean discordance score across all markers for a given method. *Given that this is a discordance score, lower values indicate better agreement across slides*. (**B**) Otsu thresholds were calculated across slides for each marker to determine marker positive cells, which were then compared to the manual labels for the markers CD3 and CD8 to determine the accuracy of defining a cell as marker positive. This is presented as the accuracy rate of recapitulating the ground truth labels—*given that this is a measurement of accuracy, higher values indicate better agreement between the normalized data and labels*. Note also that for each of these plots, the top row indicates the results from the raw, unadjusted data

In order to quantify how the normalization methods impact cell classification, we compared Otsu thresholding estimated at the slide level and across slides for each method to generate a discordance score and compare this to raw data (Fig. 2A). Compared to the epithelium/stromal markers in the dataset, less identifiable markers like CD3 and CD8 yield the worst performance across nearly all methods, with large increases in the discordance score. Most methods increase the mean discordance score relative to the unadjusted data, with the exception of the mean division, mean division $\;{\text{log}}_{10}$ and the mean division $\;{\text{log}}_{10}$ with functional data registration. This evaluation again aligns with earlier assessments and suggests that these methods present improvements in the slide-to-slide agreement across all markers compared to the unadjusted data. We also observe that when comparing threshold discordance scores across all markers, these three methods yield the lowest values and are the only methods to reduce this rate relative to the raw data (Table 2).

#### 3.1.3 Proportions of variance

**Fig. 3.**
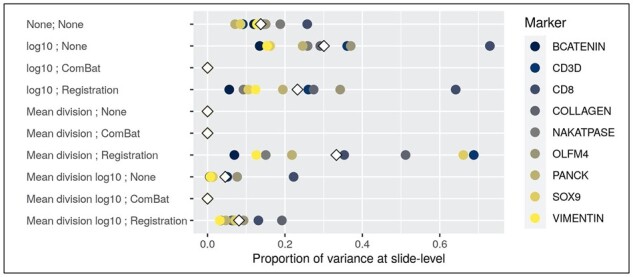
Proportion of variance present at slide-level in random effects model. Scatter plots that denote the proportion of variance at the slide-level for each normalization method for each of the marker channels in this dataset. Variance proportions were calculated using a random effects model with a random intercept for slide—methods that perform well should reduce the slide level variance. Note also that the top row indicates the results from the raw, unadjusted data

To understand how well each method removes slide-related variability, we fit a random effects model on the median cell intensities after applying each combination of transformation and normalization. The ComBat algorithm, by design, removed all of the variability related to slide across all methods (Fig. 3, Table 2). The only other method that entirely removes all slide-to-slide variance across all marker channels is the mean division method—for the mean division $\;{\text{log}}_{10}$ and mean division $\;{\text{log}}_{10}$ with functional data registration methods, we also observe reduction in variance (though not completely removed) relative to the unnormalized data. And while ComBat reduces slide variability, it completely removes slide effects that may include biological differences. In short, the results of this metric suggest the utility of the mean division methods in removing slide-level variance across marker channels.

### 3.2 Preservation of existing biological signal

#### 3.2.1 Marker-positive accuracy using Otsu thresholds

We further utilized Otsu thresholding to identify marker positive cells and compared these to the manual labels for CD3 and CD8 to determine which normalization methods most accurately recapitulate the raw data (Fig. 2B). Results suggest that the scale of the data is pivotal in whether a method maintains marker-positive accuracy, with each of the methods on the $\;{\text{log}}_{10}$ scale demonstrating dramatic reductions in marker-positive accuracy compared to the raw data, while the mean division method performs the best across all methods. The methods that have performed well in the aforementioned evaluation metrics perform well here, namely the mean division method and the mean division $\;{\text{log}}_{10}$ with functional data registration. This continues to suggest these methods reduce the slide-to-slide variation present in the data while accurately capturing marker-positive cells after transformation.

#### 3.2.2 UMAP embedding

**Fig. 4.**
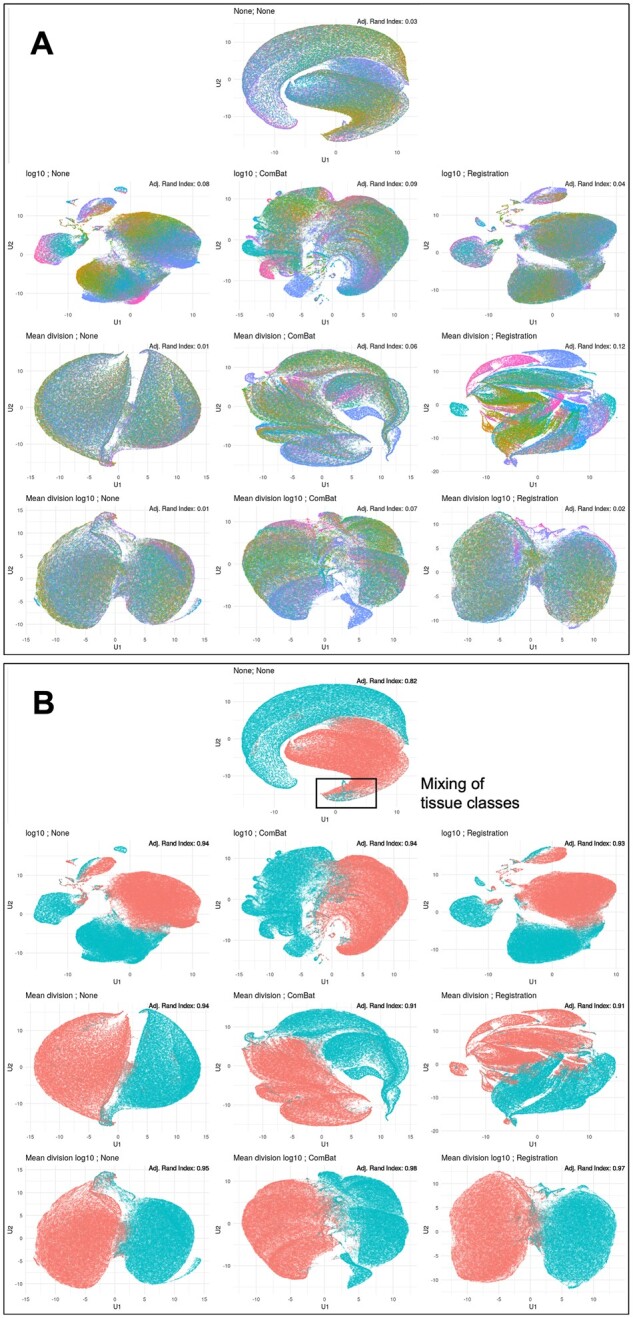
UMAP embedding of data for each transformation method. UMAP embedding of the transformed data with points colored by slide identifier (**A**) and tissue type (**B**). The rectangle in (B) denotes the mixing of tissue classes present in the raw, unadjusted data UMAP embedding. Adjusted Rand index values for each embedding are presented in the top right corner

We compared UMAP embeddings of four related markers across normalization methods to compare the separation of epithelium and stromal tissue labels. In the raw data, the embeddings separate well (Adj. Rand Index: 0.82); however, the data includes the presence of outliers that suggest mixing of the tissue classes in the UMAP embedding space (Fig. 4A). Nearly all methods implemented improve upon the separation of groups based on the adjusted Rand index, yet many of these methods present co-localization that does not clearly depict separation as desired. We do observe distinct separation of the aforementioned methods of interest: mean division (Adj. Rand Index: 0.94), mean division $\;{\text{log}}_{10}$ (Adj. Rand Index: 0.95) and the mean division $\;{\text{log}}_{10}$ with functional data registration (Adj. Rand Index: 0.97)—each of these UMAP embeddings presents distinct groups that suggests these methods are improving the separation of these two tissue classes.

We also compared the distribution of the unique slide identifiers in the UMAP embeddings of these four markers, which in the raw data (Adj. Rand Index: 0.033) points to specific slide co-localization in the data (Fig. 4B, Table 2). In this case, we desire low values of the adjusted Rand index, which suggest poor prediction of slide labels and indicate the removal of slide-level variance. Many of the methods, particularly those implementing the ComBat algorithm, worsen the distribution of these slide identifiers and increase the adjusted Rand index, suggesting additional slide-to-slide noise added to the data. This suggests that ComBat removes both biological signal and slide-to-slide effects that are exaggerated in the UMAP embedding space. In contrast, there is reduced slide-to-slide clustering in the UMAP embeddings for each of the following methods: mean division (Adj. Rand Index: 0.01), mean division $\;{\text{log}}_{10}$ (Adj. Rand Index: 0.01) and mean division $\;{\text{log}}_{10}$ with functional data registration (Adj. Rand Index: 0.02). These methods appear to both reduce the observed slide-to-slide variation noted here and in the aforementioned results, while maintaining the necessary biological signal of interest.

## 4 Discussion

In this article, we derived the ComBat algorithm for a new modality and employed a novel use of functional data registration to align histograms of multiplexed imaging data. In the absence of a gold standard for comparison in multiplexed imaging data, validating any normalization procedure is challenging. The suggested evaluation framework introduced here can be used to assess the presence and reduction of slide effects in multiplexed imaging data, which we implemented to evaluate 12 combinations of transformations and normalization methods. Furthermore, our framework can be applied in the absence of a ground truth by quantifying the amount of slide-related variability and comparing to manually labeled biological features, providing a foundation for further development of evaluation criteria in the multiplexed domain. Also note that since the proposed methods are applied within a given marker channel, this work can be extended into other imaging domains like IHC that do not involve multiplexing.

Similarly, the use of Otsu thresholding in this article is the standard procedure for imaging domains like IHC (Trinh *et al.*, 2017; Tsujikawa *et al.*, 2019). However, markers like the phosphorylated epidermal growth factor receptor are typically categorized into multiple groups based on staining intensity (Hashmi *et al.*, 2018; Shan *et al.*, 2017). While the Otsu threshold may not capture this categorization, it remains a reasonable proxy for these quantitative markers in the absence of a pathologist, and other metrics implemented here like the Anderson–Darling statistic may be more appropriate. Furthermore, future methods development could focus on implementing multi-Otsu thresholding methods into the threshold discordance score, or adapt marker-specific thresholding methods that better capture variability in the quantitative markers. Notably, the correspondence between a marker positive cell defined by an Otsu threshold and biological signal is not necessarily one-to-one. For example, the $\;{\text{log}}_{10}$ transformation non-linearly compresses the domain, such that a larger proportion of the *x*-axis is allotted to cells that are marker negative (background and unexpressed cells), which may have led to greater variability in the Otsu thresholds.

We find that the raw data scale has clear slide-to-slide variation present, and that normalization methods can reduce slide level variation while preserving and improving biological signal relative to the raw, unadjusted data. These findings suggest that the mean division transformation method reduces slide variability and improves the biological signal. In addition, the mean division $\;{\text{log}}_{10}$ scale (unnormalized) performs well across all evaluation metrics, with the noted exclusion of results for the marker CD8. This discrepancy is remedied with the functional data registration, which is a limitation of the mean division $\;{\text{log}}_{10}$ transformation but points to the robustness of the registration algorithm to maintain and improve the quality of the data.

However, note that the registration algorithm does not perform well with skewed data, suggesting that improvements we see in data that appears bi-modal (e.g. better suited to the non-parametric assumption of functional data) is not necessarily transferable to right-skewed data that violates assumptions of smoothness in the B-spline basis—future work could explore this result. The ComBat method performs adequately, but appears to over normalize the data and relies heavily on a Gaussian assumption that is violated in this skewed-right dataset. The clear limitation of this normalization method and others is that when applied to whole tissue slides, any between slide variability is confounded with biological variability. Recent adaptations of ComBat like ComBat-seq for RNA-seq data may provide a better framework to implement in the multiplexed imaging space (Zhang *et al.*, 2020), including future work that could address how the algorithm handles zeroes. Note also that recent advances applying deep learning in fluorescence microscopy analysis combine information across heterogeneous combinations of markers to ameliorate similar problems that we address in this article, namely technical variation and comparing disparate data sources (Gomariz *et al.*, 2021)—this could be a valuable avenue for future normalization approaches.

In practice, the mean division method is ‘good enough’—it is simple, computationally efficient, and appears the least likely to introduce error while still reducing slide-to-slide variation and maintaining biological signal. The mean division $\;{\text{log}}_{10}$ method may be necessary in the case of statistical modeling, since skewed distributions are not suitable for many statistical models, but may not be the best way to represent cell intensities as a predictor variable (as appears the case for the mean division method). We see that in the case of mean division $\;{\text{log}}_{10}$ data, it may be necessary to use the registration algorithm to remedy discrepancies like those visible for the marker CD8.

## Funding

This work was supported by the National Institutes of Health (T32LM012412 to C.R.H., R01DK103831 and U01CA215798 to K.S.L. and U2CCA233291 to R.J.C., K.S.L. and M.J.S.), the Colorado Clinical and Translational Sciences Institute (UL1TR002535) and the Vanderbilt Ingram Cancer Center GI SPORE (P50CA236733). Study activities were conducted in part by the Survey and Biospecimen Shared Resource (P30CA68485), the Tissue Pathology Shared Resource (P30CA068485 and U24DK059637), the Digital Histology Shared Resource, the NCI Cooperative Human Tissue Network (CHTN) Western Division (UM1CA183727) and REDCap (UL1TR000445).

## Data Availability Statement

The data underlying this article are available in the HTAN Data Portal at https://data.humantumoratlas.org/.

*Conflict of Interest*: none declared. 

## Supplementary Material

btaa877_Supplementary_Data
