# Supplementary material for: Quantifying and correcting slide-to-slide variation in multiplexed immunofluorescence images
Source: Bioinformatics. 2022 Jan 4;38(6):1700–7. doi: 10.1093/bioinformatics/btab877 (PMC8896603; doi:10.1093/bioinformatics/btab877)
Supplement: btaa877_Supplementary_Data [file btaa877_supplementary_data.pdf]

Supplementary Materials for "Quantifying and correcting slide-to-slide variation in multiplexed immunofluorescence images"

Harris, C.R.[1], McKinley, E.T.[2,3], Roland, J.T.[2,4], Liu, Q.[1,5], Shrubsole, M.J.[6], Lau, K.S.[2,3], Coffey, R.J.[2,7], Wrobel, J.[8], Vandekar, S.N.[1]

(1) Department of Biostatistics, Vanderbilt University Medical Center, Nashville, TN, USA

(2) Epithelial Biology Center, Vanderbilt University Medical Center, Nashville, TN, USA

(3) Department of Cell and Developmental Biology, Vanderbilt University School of Medicine, Nashville, TN, USA

(4) Department of Surgery, Vanderbilt University School of Medicine, Nashville, TN, USA

(5) Center for Quantitative Sciences, Vanderbilt University Medical Center, Nashville, TN, USA

(6) Division of Epidemiology, Vanderbilt Ingram Cancer Center, Vanderbilt University Medical Center, Nashville, TN, USA

(7) Division of Gastroenterology, Hepatology, and Nutrition, Department of Medicine, Vanderbilt University Medical Center, Nashville, TN, USA

(8) Department of Biostatistics & Informatics, Colorado School of Public Health, Aurora, CO, USA

Supplementary Materials

Application of ComBat

Note from the **Methods** section of the paper that we have assumed that the standardized data $Z_{ic}(u) \sim N(\gamma_{ic}, \delta^2_{ic})$ with the following priors on the batch effects:

$$\gamma_{ic} \sim N(\gamma_c, \tau^2_c), \delta^2_{ic} \sim IG(\omega_c, \beta_c)$$

Recall that i denotes the slide from which the data was collected, c denotes the marker of interest, and u defines the unit of measuring intensity, which for this study is the median quantified marker intensity of the segmented cell. Note also that we defined $U_{ic} = \sum_{ic} u$, or the number of quantified cells present on a particular slide i for a given channel c.

Posterior Derivation for γ_{ic}

Using the empirical Bayes methodology, we must derive the posterior mean estimator of γ_{ic} to utilize in the ComBat model. Hence:

$$\begin{aligned}
\pi\left(\gamma_{ic}|Z_{ic}(u), \delta^2_{ic}\right) &= L\left(Z_{ic}(u)|\gamma_{ic}, \delta^2_{ic}\right) \cdot \pi(\gamma_{ic}) \\
&\propto \exp\left\{-\frac{1}{2\delta^2_{ic}}\sum_u \left(Z_{ic}(u) - \gamma_{ic}\right)^2\right\} \cdot \exp\left\{-\frac{1}{2\tau^2_c}(\gamma_{ic} - \gamma_c)^2\right\} \\
&= \exp\left\{-\frac{1}{2\delta^2_{ic}}\left(\sum_u Y^2_{ic}(u) - 2\sum_u Z_{ic}(u)\gamma_{ic} + U_{ic} \cdot \gamma^2_{ic}\right) - \frac{1}{2\tau^2_c}\left(\gamma^2_{ic} - 2\gamma_{ic}\gamma_c + \gamma^2_c\right)\right\} \\
&\propto \exp\left\{-\frac{1}{2}\left(\frac{U_{ic}\tau^2_c + \delta^2_{ic}}{\delta^2_{ic}\tau^2_c}\right)\left[\gamma^2_{ic} - 2\left(\frac{\tau^2_c \sum_u Z_{ic}(u) + \delta^2_{ic}\gamma_c}{U_{ic}\tau^2_c + \delta^2_{ic}}\right)\gamma_{ic}\right]\right\}
\end{aligned}$$

Which after we complete the square, we see this is posterior follows the Normal distribution with the following expectation:

$$E\left[\gamma_{ic}|Z_{ic}(u), \delta^2_{ic}\right] = \frac{\tau^2_c \sum_u Z_{ic}(u) + \delta^2_{ic}\gamma_c}{U_{ic}\tau^2_c + \delta^2_{ic}}$$

To derive an estimator of the batch effect parameter, we must define the following estimators of the hyperparameters:

$$\bar{\gamma}_c = \frac{1}{U_{ic}}\sum_i \hat{\gamma}_{ic} \text{ and, } \bar{\tau}^2_c = \frac{1}{U_{ic} - 1}\sum_i (\hat{\gamma}_{ic} - \bar{\gamma}_c)^2$$

Hence we now derive the following estimator of γ_{ic}:

$$\gamma^*_{ic} = \frac{\bar{\tau}^2_c U_{ic}\hat{\gamma}_{ic} + \delta^{2*}_{ic}\bar{\gamma}_c}{U_{ic}\bar{\tau}^2_c + \delta^{2*}_{ic}}$$

Posterior Derivation for δ^2_{ic}

We employ the same methodology to derive the posterior mean estimator of δ^2_{ic}:

$$\begin{aligned}
\pi\left(\delta^2_{ic}|Z_{ic}(u),\gamma_{ic}\right) &= L\left(Z_{ic}(u)|\gamma_{ic},\delta^2_{ic}\right)\cdot\pi(\delta^2_{ic}) \\
&\propto {\delta^2_{ic}}^{-\frac{U_{ic}}{2}}\exp\left\{-\frac{1}{2\delta^2_{ic}}\sum_u\big(Z_{ic}(u)-\gamma_{ic}\big)^2\right\}\cdot{\delta^2_{ic}}^{-(\omega_c+1)}\exp\left\{-\frac{\beta_c}{\delta^2_{ic}}\right\} \\
&= {\delta^2_{ic}}^{-\left(\left[\frac{U_{ic}}{2}+\omega_c\right]+1\right)}\exp\left\{-\frac{1}{2\delta^2_{ic}}\left(\sum_u Y^2_{ic}(u)-2\sum_u Z_{ic}(u)\gamma_{ic}+U_{ic}\cdot\gamma^2_{ic}\right)-\frac{\beta_c}{\delta^2_{ic}}\right\} \\
&\propto {\delta^2_{ic}}^{-\left(\left[\frac{U_{ic}}{2}+\omega_c\right]+1\right)}\exp\left\{-\frac{1}{\delta^2_{ijc}}\left(\beta_c+\frac{1}{2}\sum_u\big(Z_{ic}(u)-\gamma_{ic}\big)^2\right)\right\}
\end{aligned}$$

Which we note is an Inverse Gamma distribution with the following expectation:

$$E\left[\delta^2_{ic}|Z_{ic}(u),\gamma_{ic}\right]=\frac{\beta_c+\frac{1}{2}\sum_u\big(Z_{ic}(u)-\gamma_{ic}\big)^2}{\frac{U_{ic}}{2}+\omega_c-1}$$

To derive an estimator of the batch effect parameter, we must define the following estimators:

$$\hat{\delta}^2_{ic}=\frac{1}{U_{ic}-1}\sum_u(Z_{ic}(u)-\hat{\gamma}_{ic})^2$$

We then calculate the sample mean of the $\hat{\delta}^2_{ic}$, $\bar{M}_c$ and $\bar{S}^2_c$ and set these equal to the moments of an Inverse Gamma distribution to yield the following estimators:

$$\bar{\omega}_c=\frac{\bar{M}_c+2\bar{S}^2_c}{\bar{S}^2_c}\text{ and, }\bar{\beta}_c=\frac{\bar{M}^3_c+\bar{M}_c\bar{S}^2_c}{\bar{S}^2_c}$$

Hence we now derive the following estimator of δ^2_{ic}:

$$\delta^{2*}_{ic}=\frac{\bar{\beta}_c+\frac{1}{2}\sum_u\big(Z_{ic}(u)-\hat{\gamma}_{ic}\big)^2}{\frac{U_{ic}}{2}+\bar{\omega}_c-1}$$

CD3 and CD8 Density Plots

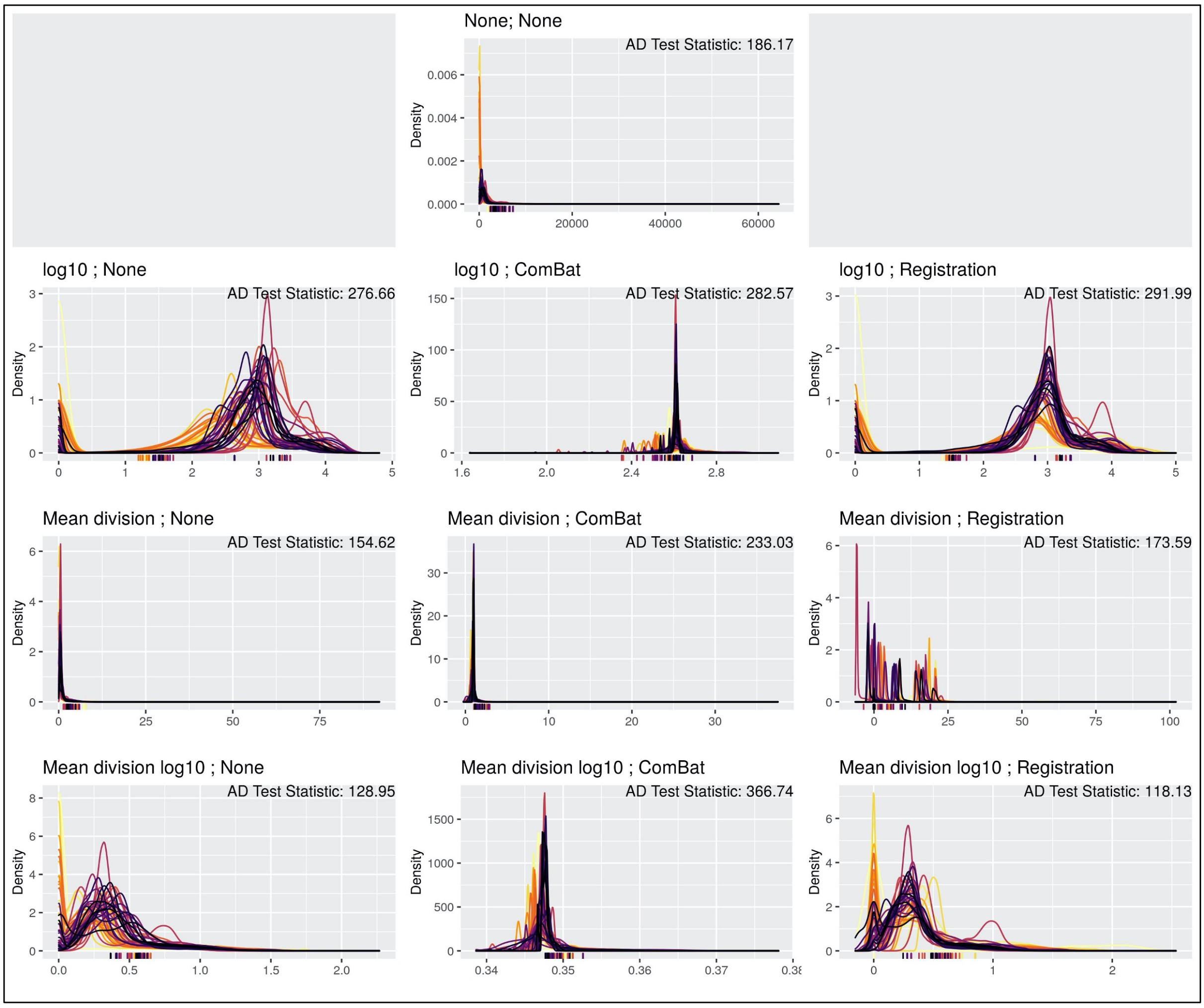

Supplementary Figure 1: Visual comparison of CD3D marker densities for each transformation method

Density plots for the median cell intensity of the marker CD3D, where each color represents a different slide in the dataset. Each row is aligned with the scale transformations present in **Table 1**, where each column also matches with the normalization algorithms in **Table 1**. The ticks on the x-axis represent the Otsu thresholds for each slide for that transformed data, where the color again corresponds to the slide (such that the colors are one-to-one between threshold and density plot). Anderson-Darling test statistics for the marker CD3D are presented for each method in the top right corner.

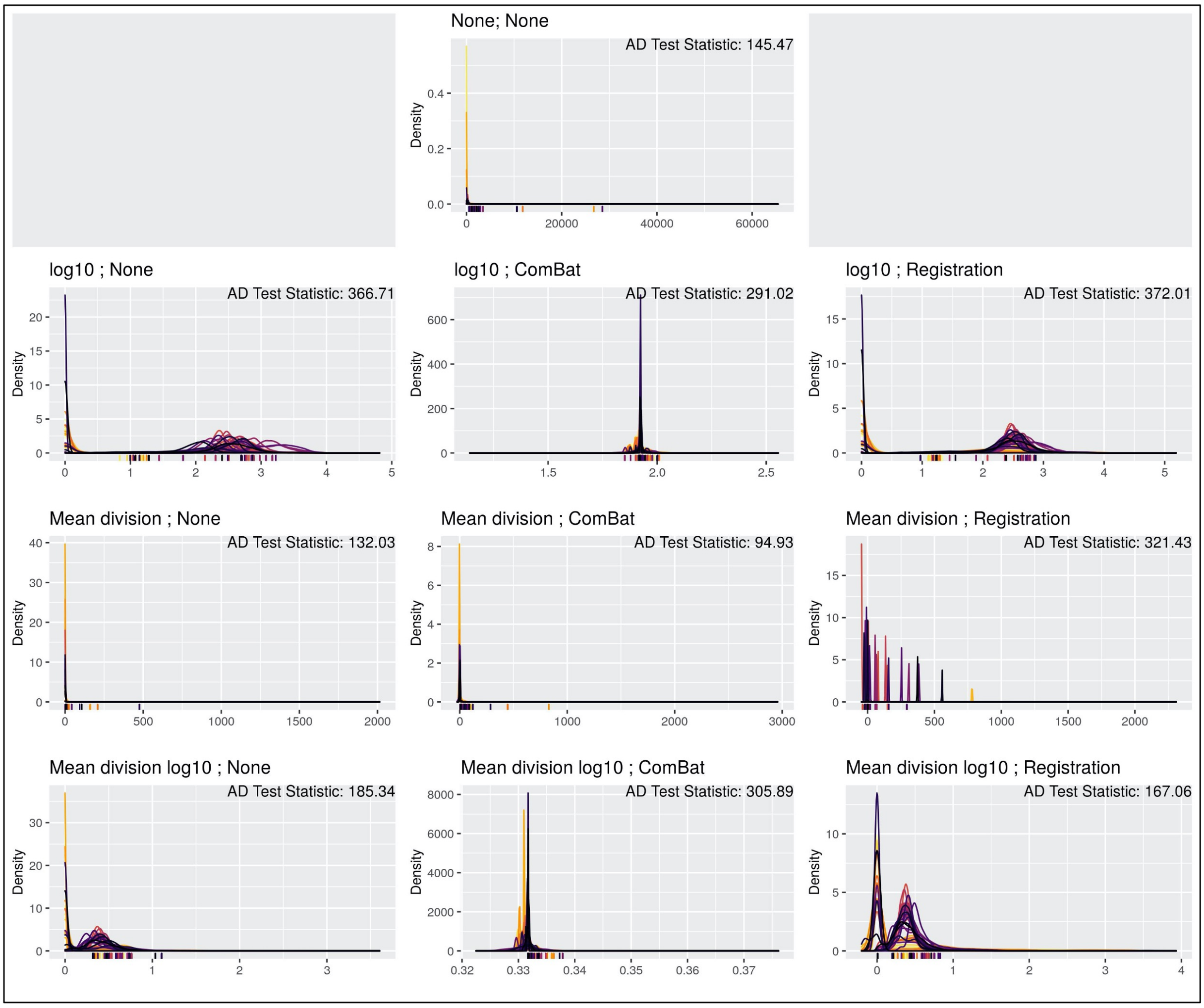

Supplementary Figure 2: Visual comparison of CD8 marker densities for each transformation method

Density plots for the median cell intensity of the marker CD8, where each color represents a different slide in the dataset. Each row is aligned with the scale transformations present in **Table 1**, where each column also matches with the normalization algorithms in **Table 1**. The ticks on the x-axis represent the Otsu thresholds for each slide for that transformed data, where the color again corresponds to the slide (such that the colors are one-to-one between threshold and density plot). Anderson-Darling test statistics for the marker CD8 are presented for each method in the top right corner.

Preservation of Cell Proportions

To measure the preservation of biological signal for each transformation method, we first quantified cell proportions in various tissue classes using Otsu thresholds. This method uses the manual labels for CD3- and CD8-positive cells to calculate the proportion of positive cells within each level of the data (e.g.

slide identifier, slide region). This metric visualizes the change in baseline cell proportions of CD3- and CD8-positive cells for each transformation algorithm implemented using raincloud plots to compare the distribution, box plot, and densities of marker positive cells.

We compared CD3 and CD8 cell proportions within epithelium and stroma using the proportions estimated by Otsu thresholding after each normalization method to quantify the preservation of biological signal for each method, as compared to those estimated using the manual labels (Supplementary Figure 3). For both CD3 and CD8, we see that the $\log_{10}$ scale does not replicate the cell proportions from the manual labels, while the mean division $\log_{10}$ performs well in both markers across normalization algorithms (again excluding the unnormalized mean division $\log_{10}$ for CD8). Per this metric in both CD3 and CD8, and re-affirming prior evaluation, the mean division method and the mean division $\log_{10}$ with functional data registration maintain the cell proportions most closely. This again points to the ability of these methods to robustly maintain biological signal in the unadjusted data while removing slide-to-slide variation.

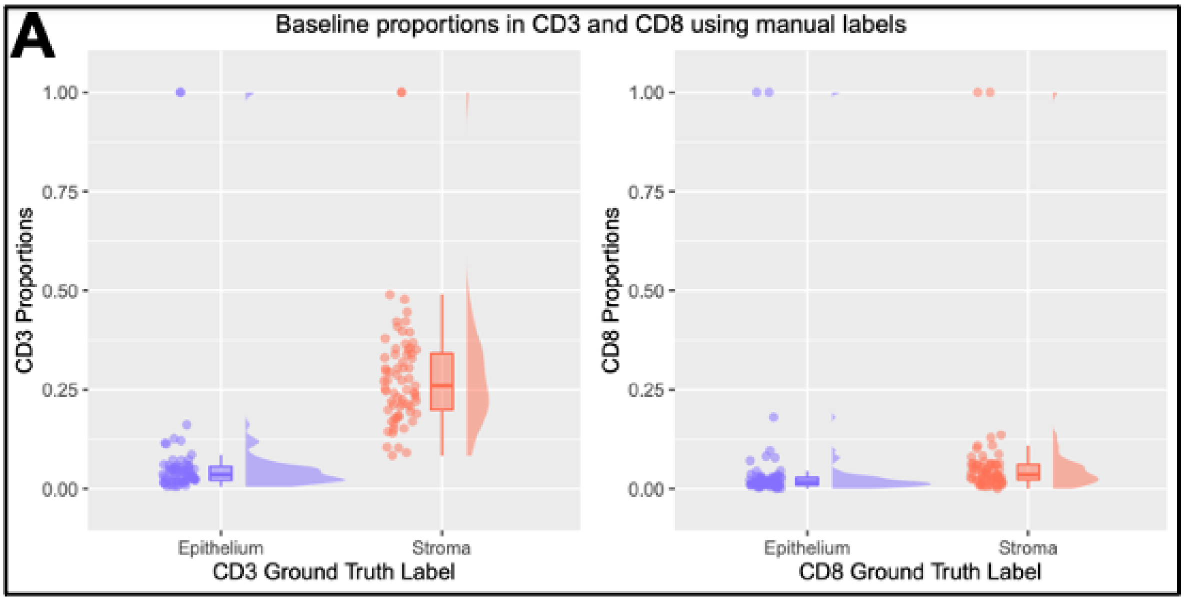

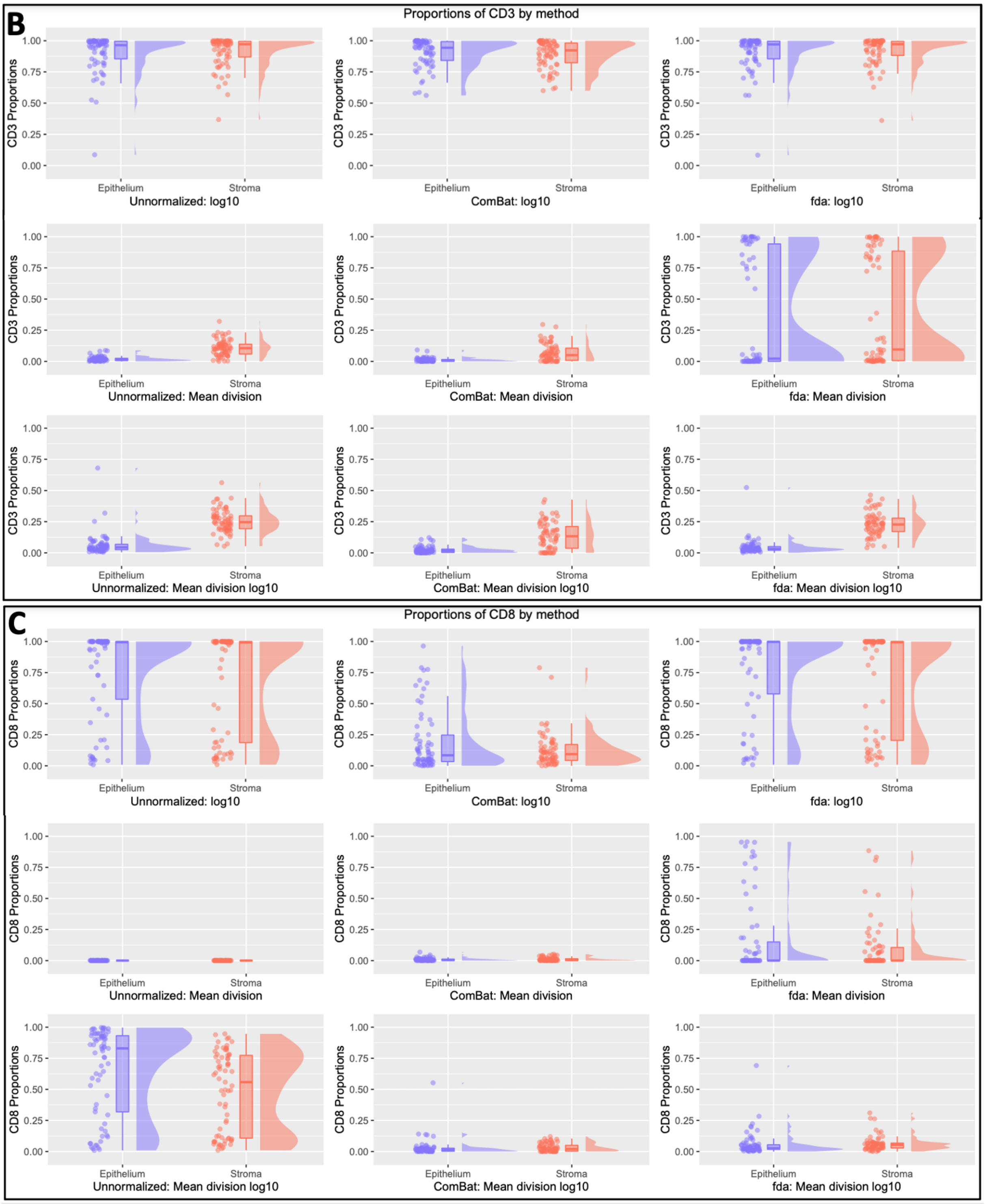

Supplementary Figure 3: Comparison of cell proportions for each transformation method A comparison of estimated proportions for the manually labeled cells for CD3 and CD8 (A) to the proportions of (B) CD3 and (C) CD8 for each normalization methods. Positive cells for the normalization methods are determined by Otsu thresholding across all slides. Methods that maintain similar estimates to the manual labels are considered more accurate.

Additional Transformations

In this section we present multiple transformations and normalization techniques that are not included in the main text. These include the following (using the same notation as Table 1):

- " $\log_{10}$ then mean division ": $\frac{\log_{10}(y+1)}{\text{mean}(\log_{10}(y+1))}$
- " $\log_{10}$ then mean subtract ": $\log_{10}(y+1) - \text{mean}(\log_{10}(y+1))$
- " $\log_{10}$ Z-transformation ": $\frac{\log_{10}(y+1)-\text{mean}(\log_{10}(y+1))}{\text{SD}(\log_{10}(y+1))}$

Here we recreate the figures and results from the main text to demonstrate the efficacy of some of these transformations. For clarity, we also include the raw, unadjusted data ("None ; None") and the best performing method, the mean division method, for comparison. In summary, we see that across the evaluation framework we introduce, none of these methods surpasses the mean division method in terms of reducing slide-to-slide variation or maintaining biological signal.

**Method**	**Mean AD Test Statistic**	**Mean Otsu Discordance Score**	**Adj. Rand Index (Slide ID)**	**Mean Variance Proportion (Slide ID)**
None; None	275.019	0.085	0.033	0.138
Mean division ; None	138.774	0.041	0.007	0.000
$\log_{10}$ then mean division ; None	212.030	0.141	0.066	0.000
$\log_{10}$ then mean subtract ; None ; None	137.474	0.088	0.052	0.000
$\log_{10}$ Z-transformation ; None ; None	131.621	0.068	0.014	0.000

Supplementary Table 1: Quantitative metrics comparing additional transformations
Results from the k-samples Anderson-Darling test statistic, the Otsu discordance score, and the variance proportion at the slide level from the random effects modeling, all averaged across marker channels, as well as the adjusted Rand index for the slide identifiers comparing the raw data to the normalized data.

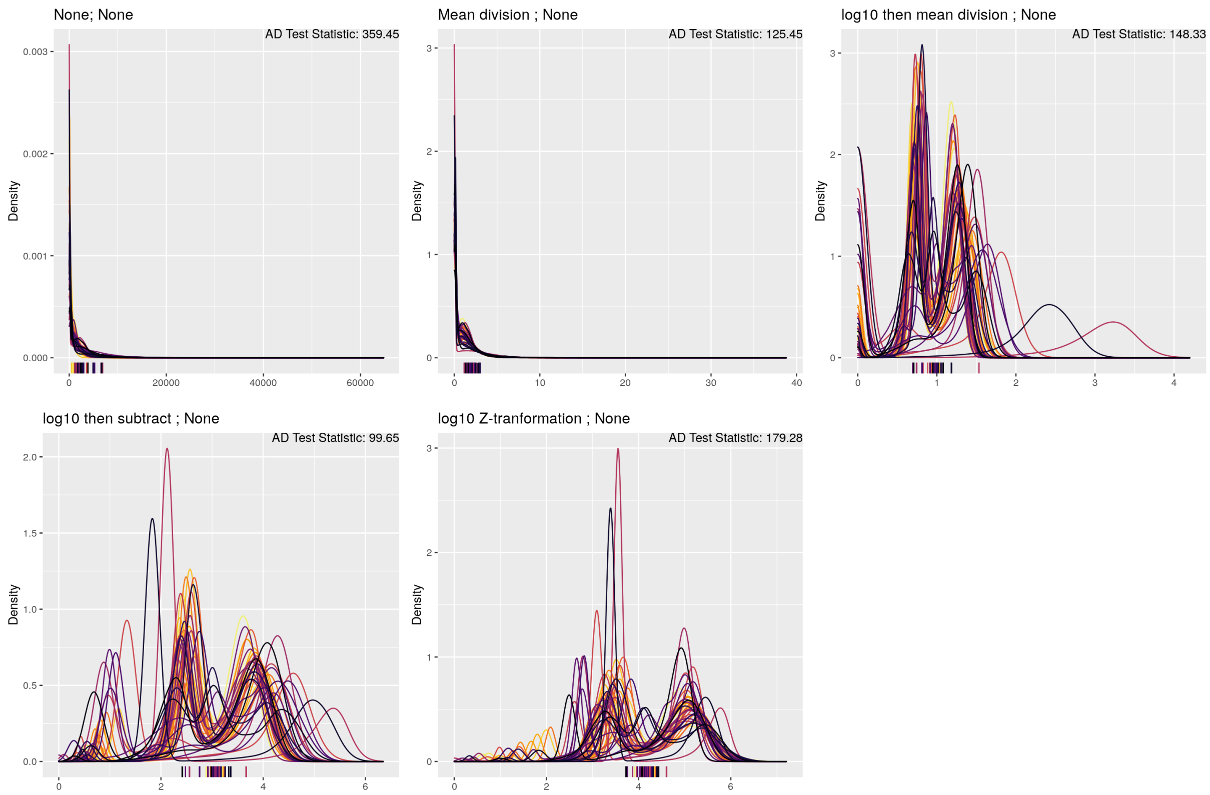

Supplementary Figure 4: Visual comparison of vimentin marker densities for each additional transformation

Recreation of **Figure 1**: Density plots for the median cell intensity of the marker vimentin, where each color represents a different slide in the dataset. Each row is aligned with the scale transformations present in **Table 1**, where each column also matches with the normalization algorithms in **Table 1**. The ticks on the x-axis represent the Otsu thresholds for each slide for that transformed data, where the color again corresponds to the slide (such that the colors are one-to-one between threshold and density plot). Anderson-Darling test statistics for the marker vimentin are presented for each method in the top right corner.

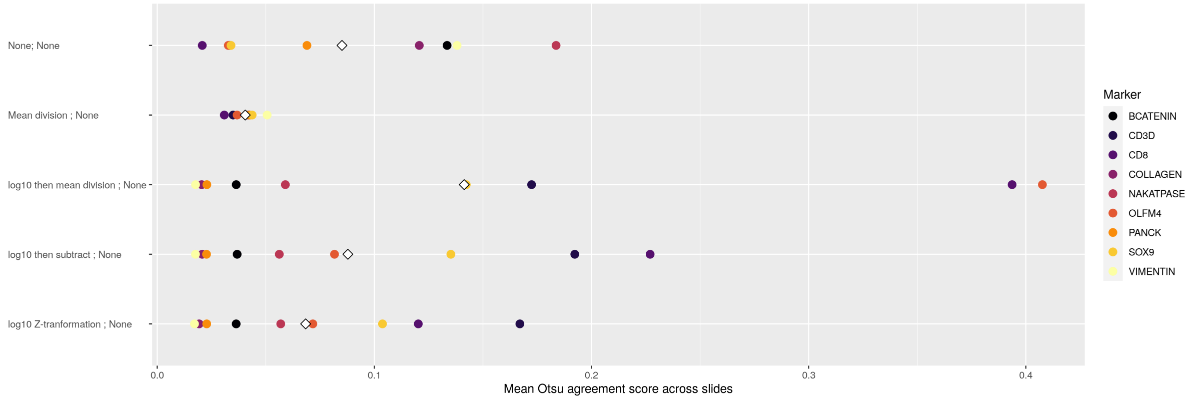

Supplementary Figure 5: Otsu discordance scores for each additional transformation

Recreation of **Figure 2A**: Otsu thresholds were calculated at the slide-level for each marker and compared to a global Otsu threshold for each marker to calculate a discordance score to compare transformation methods. The mean difference of the slide-level Otsu thresholds and the global Otsu thresold is then calculated for each marker, presented as a point for each of the 9 markers, with the white diamond representing the mean discordance score across all markers for a given method. **Given that this is a discordance score, lower values indicate better agreement across slides.**

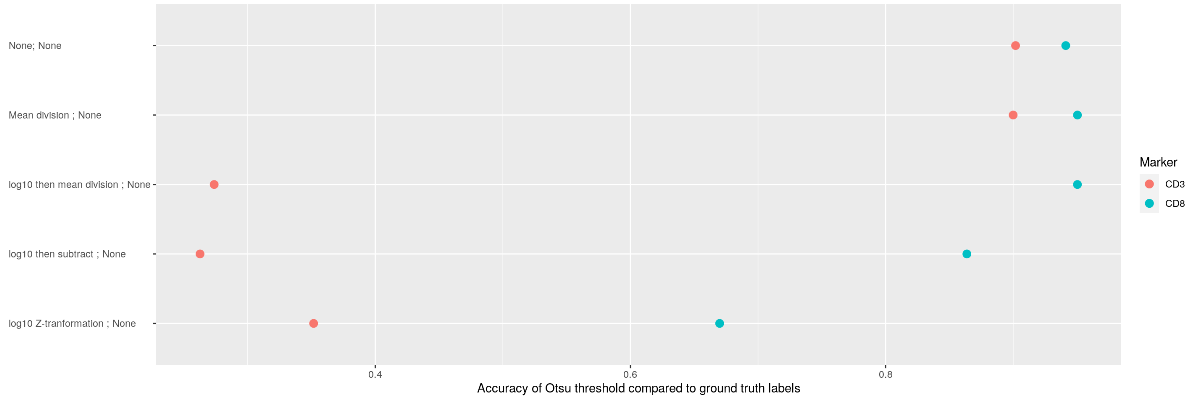

Supplementary Figure 6: Otsu accuracy for each additional transformation

Recreation of **Figure 2B**: Otsu thresholds were calculated across slides for each marker to determine marker positive cells, which were then compared to the manual labels for the markers CD3 and CD8 to determine the accuracy of defining a cell as marker positive. This is presented as the accuracy rate of recapitulating the ground truth labels - **given that this is a measurement of accuracy, higher values indicate better agreement between the normalized data and labels.**

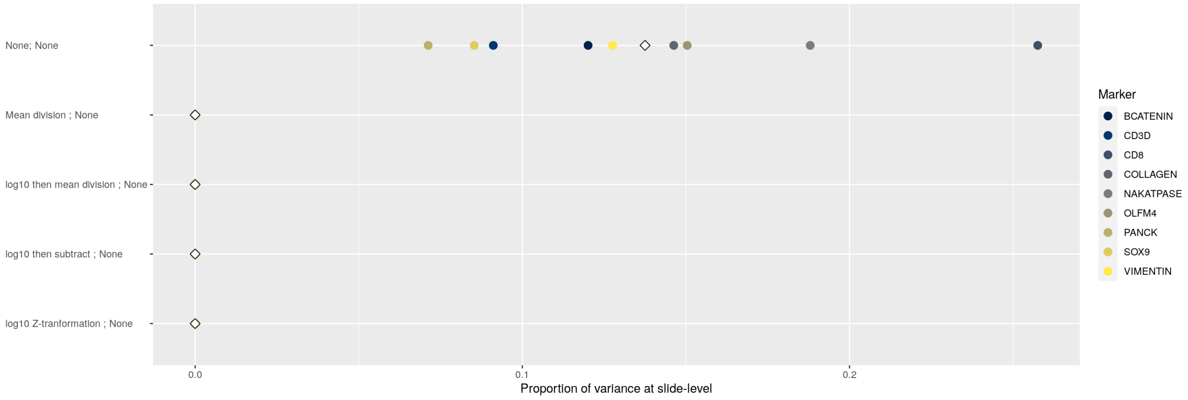

Supplementary Figure 6: Proportion of variance present at slide-level in random effects model for each additional transformation

Recreation of **Figure 3**: Scatter plots that denote the proportion of variance at the slide-level for each normalization method for each of the marker channels in this dataset. Variance proportions were calculated using a random effects model with a random intercept for slide – methods that perform well should reduce the slide level variance. Note also that the top row indicates the results from the raw, unadjusted data and the second row indicates results from the mean division method.

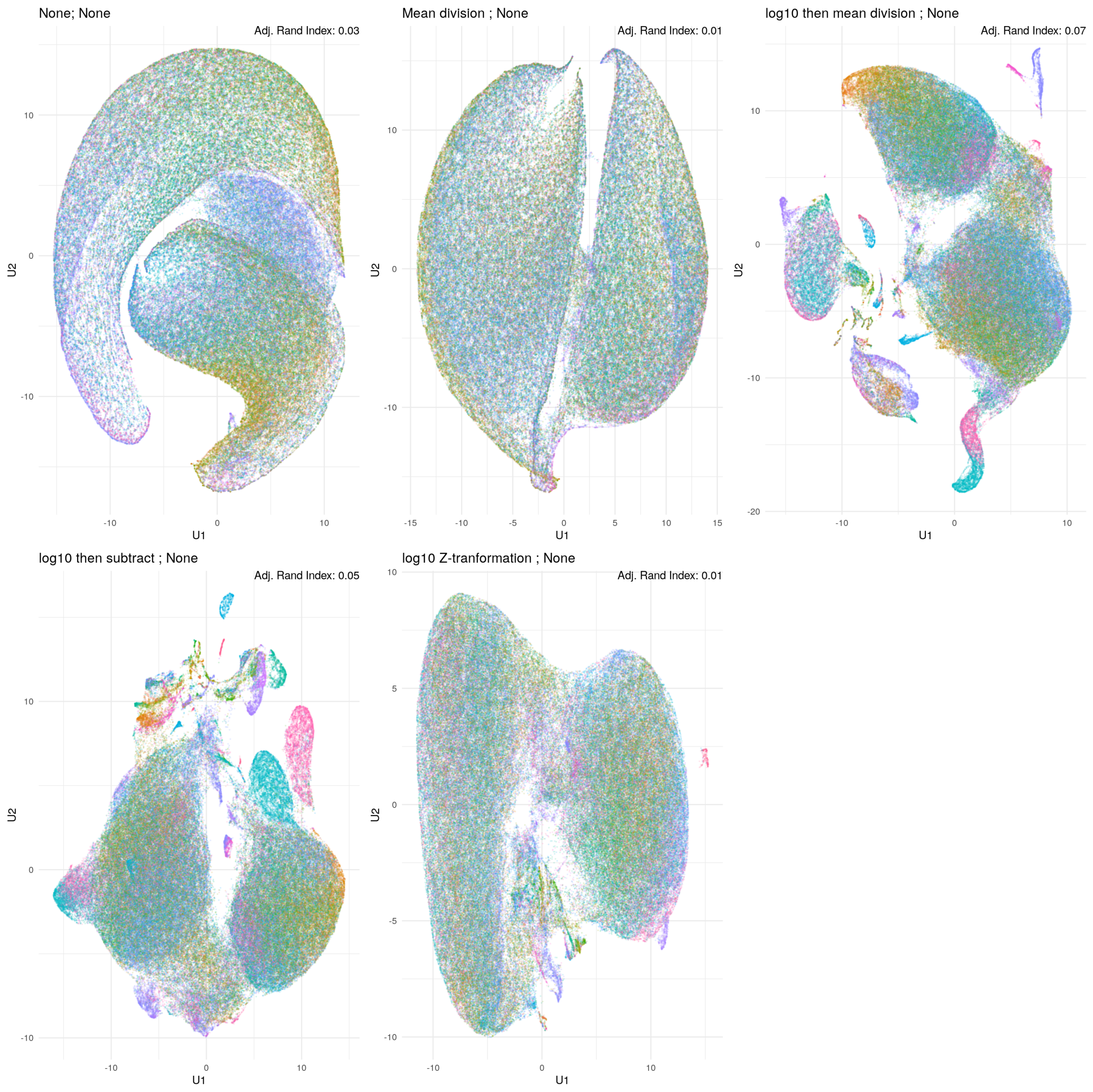

Supplementary Figure 8: UMAP embedding of data for each additional transformation (slide)

Recreation of **Figure 4A**: UMAP embedding of the transformed data with points colored by slide identifier.

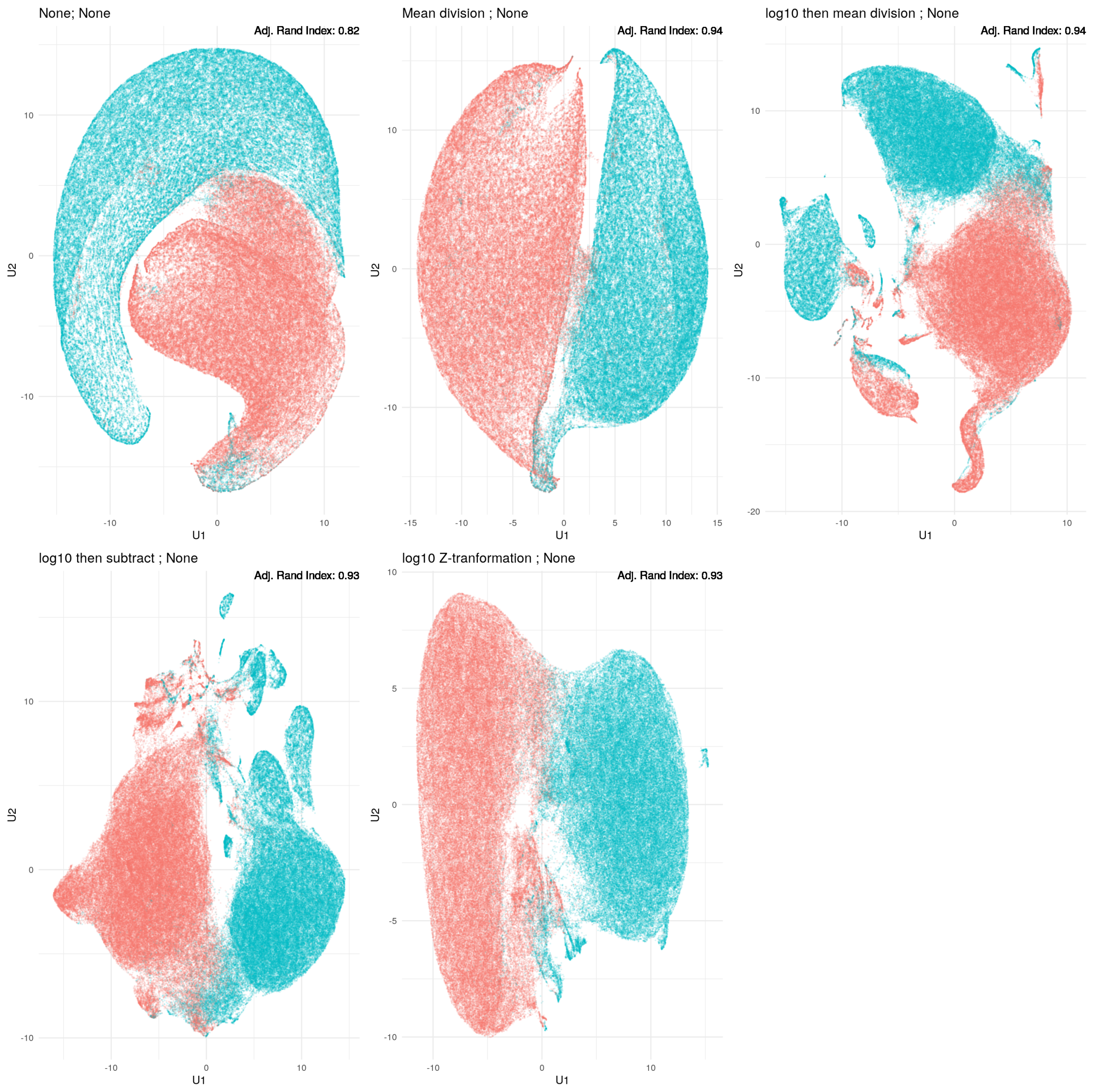

Supplementary Figure 8: UMAP embedding of data for each additional transformation (tissue)

Recreation of **Figure 4B**: UMAP embedding of the transformed data with points colored by tissue type.

**Dataset definition**	**Marker**
ACTININ	Actinin
BCATENIN	Beta Catenin
CD11B	CD11b
CD20	CD20
CD3	CD3D
CD4	CD4
CD45	Protein tyrosine phosphatase, receptor type, C
CD68	CD68
CD8	CD8
CGA	Chromogranin A
COLLAGEN	Collagen
COX2	Cyclooxygenase-II
ERBB2	Erb-B2 Receptor Tyrosine Kinase 2
FOXP3	Forkhead Box P3
HLAA	Human Leukocyte Antigen
LYSOZYME	Lysozyme
MUC2	Mucin 2
NAKATPASE	Na^+/K^+-ATPase
OLFM4	Olfactomedin 4
PANCK	Pan-Cytokeratin
PCNA	Proliferating Cell Nuclear Antigen
PEGFR	phospho-Epidermal Growth Factor Receptor
PSTAT3	Phosphorylated Signal Transducer and Activator of Transcription
SMA	α-Smooth Muscle Actin
SNA	Spherical Nucleic Acid
SOX9	SRY-Box 9
VIMENTIN	Vimentin
DAPI	$4'$,6-diamidino-2-phenylindole
CD45B	CD45RB
GACTIN	Globular Actin
PDL1	Programmed Death-Ligand 1
CDX2	Caudal-type Homeobox 2
MUC5AC	Mucin 5AC

Supplementary Table 2: List of markers available in the dataset.
